# The threshold of hypothyroidism after radiation therapy for head and neck cancer: a retrospective analysis of 116 cases

**DOI:** 10.1093/jrr/rrv006

**Published:** 2015-03-27

**Authors:** Masayuki Fujiwara, Norihiko Kamikonya, Soichi Odawara, Hitomi Suzuki, Yasue Niwa, Yasuhiro Takada, Hiroshi Doi, Tomonori Terada, Nobuhiro Uwa, Kosuke Sagawa, Shozo Hirota

**Affiliations:** 1 Department of Radiology, Hyogo College of Medicine, Nishinomiya, Hyogo, Japan; 2 Department of Radiation Oncology, Meiwa Cancer Clinic, Nishinomiya, Hyogo, Japan; 3 Department of Otolaryngology, Hyogo College of Medicine, Nishinomiya, Hyogo, Japan

**Keywords:** hypothyroidism, head and neck cancer, radiation therapy, late radiation toxicity

## Abstract

The purpose of the present study was to determine the risk factors for developing thyroid disorders based on a dose–volume histograms (DVHs) analysis. Data from a total of 116 consecutive patients undergoing 3D conformal radiation therapy for head and neck cancers was retrospectively evaluated. Radiation therapy was performed between April 2007 and December 2010. There were 108 males and 8 females included in the study. The median follow-up term was 24 months (range, 1–62 months). The thyroid function was evaluated by measuring thyroid-stimulating hormone (TSH) and free thyroxine (FT4) levels. The mean thyroid dose, and the volume of thyroid gland spared from doses ≥10, 20, 30 and 40 Gy (VS10, VS20, VS30 and VS40) were calculated for all patients. The thyroid dose and volume were calculated by the radiotherapy planning system (RTPS). The cumulative incidences of hypothyroidism were 21.1% and 36.4% at one year and two years, respectively, after the end of radiation therapy. In the DVH analyses, the patients who received a mean thyroid dose <30 Gy had a significantly lower incidence of hypothyroidism. The univariate analyses showed that the VS10, VS20, VS30 and VS40 were associated with the risk of hypothyroidism. Hypothyroidism was a relatively common type of late radiation-induced toxicity. A mean thyroid dose of 30 Gy may be a useful threshold for predicting the development of hypothyroidism after radiation therapy for head and neck cancers.

## INTRODUCTION

In Japan, ∼24 000 people are diagnosed with head and neck cancer annually. Radiation therapy plays an important role during the treatment of head and neck cancers. Hypothyroidism is one of the late adverse effects after radiation therapy for head and neck cancers. The mechanisms underlying the development of thyroid disorders after radiation therapy have been unclear [1].

In recent literature, the incidence of hypothyroidism ranges from 17–47.7% [2–17].

Three-dimensional conformal radiation therapy (3D-CRT) and intensity-modulated radiation therapy (IMRT) are now being performed globally, and 3D radiation treatment planning systems (RTPS) are widely used, thus making it possible to determine the estimated dose for the targets and the organs at risk.

There have been various reports about risk factors related to the dose–volume relationship for the thyroid gland [3–9], but the positive predictors were unclear, and the Quantitative Analysis of Normal Tissue Effects in the Clinic reviews did not include information about the thyroid gland.

In our institute, we have routinely examined the thyroid function of the patients who received radiation therapy for head and neck cancers since 2007, and patients who had subclinical hypothyroidism and increased serum TSH levels above 10 µU/ml were considered to require hormone-replacement therapy, based on the American Association of Clinical Endocrinologists and The American Thyroid Association (ATA/AACE) Guideline for hypothyroidism [18].

The purpose of the present study was to estimate the incidence of thyroid disorders after radiation therapy for head and neck cancers, and to determine the threshold for the development of hypothyroidism after radiation therapy based on an analysis of the dose–volume histograms (DVHs).

## MATERIALS AND METHODS

### Patient characteristics

The data from 116 patients who received radiation therapy for cytologically or histologically confirmed head and neck cancers treated with 3D-CRT in our institute from April 2007 to December 2010 were retrospectively reviewed in this study. Informed consent was obtained from all patients.

A total of 108 males and 8 females were included, and the median age of the subjects was 69 years old (range, 20–88 years old). The median follow-up duration after radiation therapy was 24 months (1–62 months). Of the 116 patients, 15 (12.9%) were excluded from the evaluation of post radiation therapy hypothyroidism because they had subclinical hypothyroidism before the start of radiation therapy. The patient characteristics are summarized in Table 1.

**Table 1.** The characteristics of the 116 patients with head and neck cancers who received radiation therapy between April 2007 and December 2010

Age (years)	20–88 (median: 69)				
Gender (male/female)	108/8				
Primary site and clinical stage					
	I	II	III	IV	total
glottis	37	9	2	1	49
supra-glottis	4	1	6	4	15
nasopharynx	2	1	3	2	8
oropharynx	0	2	6	11	19
hypopharynx	6	1	8	10	25
Thyroid volume before treatment	5.17–33.72 cm^3^ (mean: 13.38)				
Follow-up term after treatment	1–62 months (median: 24)				

The mean volume of the thyroid gland before radiation therapy calculated by the radiotherapy planning system (RTPS) was 13.38 ml (5.17–33.72 ml).

The thyroid function was evaluated by measuring the serum TSH and FT4 levels. Both of the TSH and FT4 levels were monitored in patients every 2 or 3 months after the completion of radiation therapy for the first 6 months, and then every 3–6 months thereafter and also when clinically indicated. We defined hypothyroidism as a serum TSH value greater than our institutional upper limit of normal (>5.0 µU/ml, based on the Colorado thyroid disease prevalence survey) [19]. The time to onset of thyroid dysfunction was defined as the interval between the end of radiation therapy and the first abnormal TSH level.

### Radiation therapy

All patients received 3D-CRT with conventional fractionation of 2 Gy per fraction. A total of 104 patients received 66 Gy, 12 patients received <66 Gy, but all patients received at least 60 Gy. A 4-MV photon beam was used for all patients. The 67 patients in the ‘elective node irradiation (ENI) group’ were treated with wide local fields or whole-neck fields that included ENI. On the other hand, the 49 patients in the ‘no ENI group’ were treated with localized fields for the larynx without ENI. The patients in the ENI group received 40–50 Gy for the wide local field or whole-neck field, and then received a boost for the involved field, including the gross tumor. None of the patients underwent IMRT.

### Dosimetric analysis

3D treatment plans were available for all of the 116 patients for the retrospective analysis. The mean thyroid dose and volume of the thyroid gland spared from doses ≥ 10 Gy, 20 Gy, 30 Gy, 40 Gy, 50 Gy and 60 Gy (VS10, 20, 30, 40, 50 and 60, respectively) were calculated from the dose–volume histograms (DVHs) based on the RTPS.

### Statistical analysis

The prevalence of hypothyroidism before treatment, the incidence of hypothyroidism induced by radiation therapy, and the incidence of hormone-replacement therapy were calculated using the Kaplan–Meier method.

The correlation between the development of hypothyroidism and clinical factors (age, gender, thyroid volume before treatment, co-morbidities, radiation field and combined treatment modalities) were evaluated using univariate and multivariate logistic regression analyses.

The patient age, gender, thyroid volume, comorbidities, radiation field and other treatment modalities that included neck dissection (ND) and chemotherapy (concurrent and/or sequential) were examined as grouped variables for this evaluation: ≤69 years old vs >70 years old, male vs female, ≤13.40 cm^3^ vs >13.40 cm^3^, with comorbidity vs without any comorbidity, ENI group vs no ENI group, ND group vs no ND group, and with chemotherapy vs without chemotherapy.

The correlation between the development of hypothyroidism and the dose–volume parameters for the thyroid gland was evaluated using the Kaplan–Meier method and the log-rank test. In order to evaluate the impact of the mean thyroid dose, patients were grouped as follows: <30 Gy, <40 Gy, <50 Gy and ≥50 Gy.

The mean thyroid dose, VS10, VS20, VS30, VS40, VS50 and VS60 were evaluated in the two groups (hypothyroidism group and euthyroidism group) using the *t*-test.

## RESULTS

### Incidence of hypothyroidism

A total of 39 (38.6%) of the 101 patients who had euthyroidism prior to treatment developed hypothyroidism due to radiation therapy; 21 patients (20.8%) developed hypothyroidism with decreased serum free T4 levels, and 21 patients (20.8%) required thyroid hormone replacement with levothyroxine. Eight patients who had nasopharyngeal carcinoma were included in the present study. They underwent irradiation of the pituitary region. The mean dose for the pituitary gland ranged from 4.8 Gy to 60.6 Gy, but no patients investigated in this study developed pituitary hypothyroidism.

**Fig. 1.**
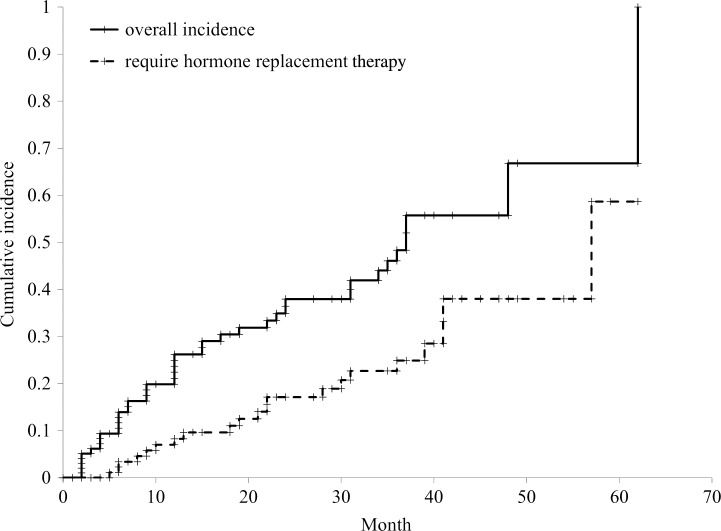
The cumulative incidence of hypothyroidism (*n* = 101). The overall incidence at one year was 21.1%, at two years was 36.4% and at three years was 48.3%. The incidence of patients who required hormone replacement at one year was 8.3%, at two years was 17.1% and at three years was 24.9%.

The cumulative incidences of hypothyroidism at one, two and three years after radiation therapy were 21.1%, 36.4% and 48.3% (Fig. 1). The cumulative incidences of patients who received thyroid hormone replacement with levothyroxine at one, two and three years after radiation therapy were 8.3%, 17.1% and 24.9% (Fig. 1). The median time to the onset of hypothyroidism was 21 months (5–57 months) after radiation therapy.

### The correlations between hypothyroidism and clinical factors

Table 2 shows the correlations between hypothyroidism and the clinical factors. In the univariate analysis, the presence of ENI and a combined treatment modality had a statistically significant impact on the development of hypothyroidism. According to the multivariate analysis, the incidence of hypothyroidism in the patients in the ENI group was significantly higher than that of the patients in the no ENI group.

**Table 2.** The correlation between the development of hypothyroidism after radiation therapy and clinical factors (*n* = 101) (Cox regression model)

			Univariate	Multivariate
Age	≤69 years old	>70 years old	N.S.	N.S.
	43.6%	34.8%		
Gender	Male	Female	N.S.	N.S.
	37.6%	62.5%		
Thyroid volume	≤13.40 cm^3^	>13.40 cm^3^	N.S.	N.S.
	48.0%	31.7%		
Comorbidity	(+)	(−)	N.S.	N.S.
	39.1%	40.6%		
Radiation field	ENI group	No ENI group	*P* < 0.05	*P* < 0.05
	42.5%	21.4%		
Neck dissection	(+)	(−)	N.S.	N.S.
	50.0%	38.2%		
Chemotherapy (concurrent and/or sequential)	(+)	(−)	*P* < 0.05	N.S.
	49.2%	23.7%		

The actual incidence of hypothyroidism in patients with a radiation field including ENI was significantly higher than that in patients without ENI in the multivariate analysis.

### The correlation between hypothyroidism and dose–volume parameters of the thyroid gland

Table 3 shows the correlations between hypothyroidism and the dose–volume parameters of the thyroid gland. The mean thyroid dose, VS10, VS20, VS30, VS40, VS50 and VS60 of the euthyroidism group were 36.83 Gy, 4.27 cm^3^, 5.07 cm^3^, 5.59 cm^3^, 6.23 cm^3^, 10.43 cm^3^ and 12.81 cm^3^, respectively, while the mean thyroid dose, VS10, VS20, VS30, VS40, VS50 and VS60 of the hypothyroidism group were 44.39 Gy, 1.28 cm^3^, 1.56 cm^3^, 1.80 cm^3^, 2.27 cm^3^, 8.56 cm^3^ and 11.19 cm^3^, respectively.

**Table 3.** A comparison of the dose and spared volume between the euthyroidism group (*n* = 62) and the hypothyroidism group (*n* = 39) (*t*-test)

	Euthyroidism	Hypothyroidism	*P*-value
Mean dose (cGy)	3683.8	4439.9	*P* < 0.05
VS10 (cm^3^)	4.27	1.28	*P* < 0.05
VS20 (cm^3^)	5.07	1.56	*P* < 0.05
VS30 (cm^3^)	5.59	1.80	*P* < 0.05
VS40 (cm^3^)	6.23	2.27	*P* < 0.05
VS50 (cm^3^)	10.43	8.56	N.S.
VS60 (cm^3^)	12.81	11.19	N.S.
Thyroid volume (cm^3^)	15.84	13.44	N.S.

VS*x*: The volume of the thyroid gland spared from doses ≥*x* Gy.

The mean dose of the hypothyroidism group was significantly higher than that of the euthyroidism group, and VS10, VS20, VS30 and VS40 of the hypothyroidism group were significantly smaller than those of the euthyroidism group.

The mean thyroid dose in the hypothyroidism group was significantly higher than that of the euthyroidism group, and the VS10, VS20, VS30 and VS40 in the hypothyroidism group were significantly smaller than those in the euthyroidism group.

**Fig. 2.**
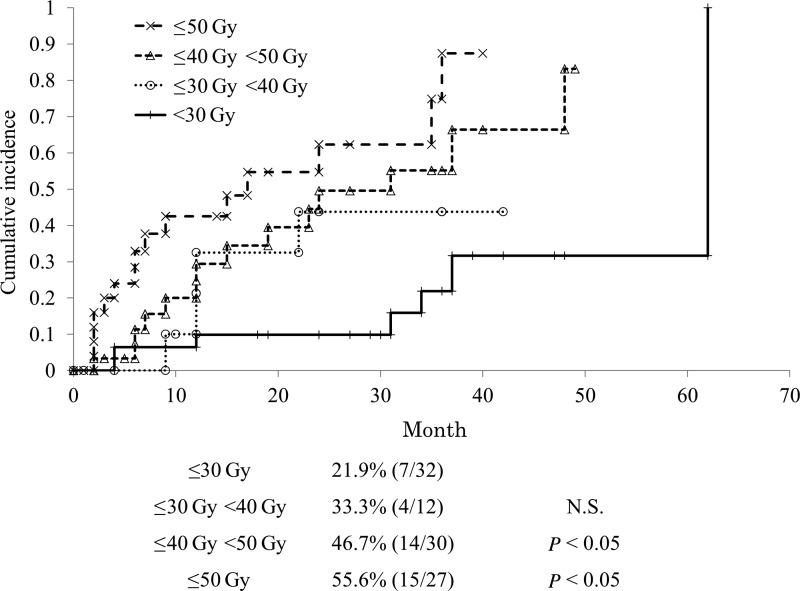
The cumulative incidence of hypothyroidism classified by the mean dose to the thyroid gland (*n* = 101). Patients who received a mean thyroid dose <30 Gy had a significantly lower incidence of hypothyroidism than those who received a higher dose.

The incidence of hypothyroidism in the <50 Gy group and the ≥50 Gy group were significantly higher than the incidence in the <30 Gy group. On the other hand, the incidence in the < 40 Gy group was higher than the incidence in the <30 Gy group, but the difference was not statistically significant (Fig. 2).

## DISCUSSION

Hypothyroidism is a well-known late toxicity after radiation therapy for head and neck cancer. The incidences that have been reported range from 17–47.7% [2–17].

The incidence of hypothyroidism after radiation therapy in the present study was comparable with that of the previous reports, and there was an increase in the incidence of hypothyroidism over time for at least three years after radiation therapy. The mechanisms underlying the development of thyroid disorders after radiation therapy are thought to include vascular damage, capsular fibrosis, iodine insufficiency due to an inadequate diet, and direct parenchymal damage [1], but the details remain unclear [1].

In the normal population, the prevalence of subclinical hypothyroidism was reported to range from 1–10% [20, 21], and 10.6% of males and 12.3% of females met the criteria for subclinical hypothyroidism in elderly people aged 70 to 79 years [22]. In Japan, the prevalence of subclinical hypothyroidism in general health check-ups was 5.1% from April 2007 to February 2011 [23]. In the present study, the prevalence of subclinical hypothyroidism was 12.9% prior to treatment. The patients who were reviewed in the present study were mostly male, but there was a high prevalence of subclinical hypothyroidism. The main cause of hypothyroidism is Hashimoto's disease, but there was only one patient who had Hashimoto's disease in these patients. Some authors have previously reported that a sudden iodine overload blocks hormone synthesis (Wolff–Chaikoff effect) [24, 25]. In the present study, most patients had undergone contrast-enhanced computed tomography studies using an iodine-containing contrast agent prior to treatment, and might have been exposed to an iodine excess. An iodine excess due to the injection of an iodine-containing contrast agent might have correlated with the high prevalence of hypothyroidism in our study. However, we could not confirm this, and the reason why there was high prevalence of subclinical hypothyroidism in this population remains unknown.

There have been various reports concerning the risk factors for hypothyroidism after radiation therapy. A combination of surgery and radiation therapy was previously reported to be a risk factor [7]. Moreover, various other risk factors (such as age [26], gender [26, 27], the radiation field [7, 10], chemotherapy [11], the period after radiation therapy [7] and smoking [11]) have been reported. In our study, only the radiation field that included ENI (such as in whole-neck irradiation) had a significant correlation with the development of hypothyroidism.

Risk factors concerning dose–volume parameters for the thyroid gland have been reported in various studies. For example, Emami *et al*. reported that the risk of hypothyroidism was estimated to be 8% with 45 Gy if the whole thyroid gland was irradiated [6]. Grande *et al*. reported that 22.4% and 56% of patients who received <60 Gy and >60 Gy to the thyroid region developed hypothyroidism [7]. Kim *et al*. reported that the thyroid V45 value can predict the development of hypothyroidism, and a V45 of 50% can be used as a threshold in RT planning [9]. Yoden *et al*. reported the percentage volume of the thyroid gland receiving radiation doses, and found that the V10 (thyroid volume receiving >10 Gy), V20 and V30 had a significant impact on the peak level of TSH [3]. Similarly, Cella reported that the V30 predicts the risk of developing hypothyroidism after sequential chemo-radiotherapy for Hodgkin's lymphoma [4]. Rønjom *et al*. reported that the risk of hypothyroidism is significantly associated with low pretreatment thyroid volumes and the mean thyroid dose [5]. Diaz *et al*. reported the impact of IMRT with thyroid dose constraints, and they recommended reducing the dose and volume of radiation to the thyroid by use of IMRT [8].

In the present study, the patients who received a mean thyroid dose <30 Gy had a significantly lower incidence of hypothyroidism than those who received higher doses. Thus, the mean thyroid dose may be a predictor of the development of hypothyroidism after radiation therapy for head and neck cancers, and a mean thyroid dose of 30 Gy may be a threshold for hypothyroidism. While the VS10, 20, 30 and 40 may also be predictors of hypothyroidism, these were not found to be significant predictive values for the development of hypothyroidism according to our multivariate analysis.

Lo Galbo *et al*. reported that the presence of thyroglobulin antibodies had a strong association with hypothyroidism after treatment for head and neck cancers [17]. An autoimmune reaction is thought to have some association with hypothyroidism, but we could not determine whether there was any association between the autoimmune reaction and hypothyroidism in the present study. Some patients who received a low radiation dose and had a small area of thyroid gland included in the radiation field also developed hypothyroidism. In these patients, autoimmune reactions may have been involved in the hypothyroidism.

There are several possible limitations associated with our study, including lack of coherence in the patient characters and the relatively short follow-up duration. Patients with a range of characteristics were included in this study, including treatment with a variety of regimens of chemotherapy and/or fields for radiation therapy. The absorbed dose of the thyroid gland was not uniform during the series of irradiation because of the shrinking radiation field. Also, the duration of follow-up was not long enough to thoroughly evaluate late toxicities. Nevertheless, our study provides useful information about the risk of hypothyroidism after radiation therapy for head and neck cancers, thus may be useful for both treatment planning and the follow-up after treatment.

In conclusion, hypothyroidism after radiation therapy was found to be a relatively common late radiation toxicity. Patients who have received radiation therapy for head and neck cancers may require thyroid function screening after radiation therapy.

The present results suggest that the incidence of hypothyroidism may depend on the absorbed dose for the thyroid gland. The mean thyroid dose may predict the risk of developing hypothyroidism after conformal radiation therapy for head and neck cancers, and a mean thyroid dose of 30 Gy may be a useful threshold for determining the dose constraint during treatment planning. Further investigations with a longer-term follow-up and larger series are needed.
